# The Transcription Factor SOX18 Regulates the Expression of Matrix Metalloproteinase 7 and Guidance Molecules in Human Endothelial Cells

**DOI:** 10.1371/journal.pone.0030982

**Published:** 2012-01-23

**Authors:** Martina Hoeth, Heide Niederleithner, Renate Hofer-Warbinek, Martin Bilban, Herbert Mayer, Ulrike Resch, Christof Lemberger, Oswald Wagner, Erhard Hofer, Peter Petzelbauer, Rainer de Martin

**Affiliations:** 1 Department of Vascular Biology and Thrombosis Research, Medical University of Vienna, Vienna, Austria; 2 Department of Dermatology, Medical University of Vienna, Vienna, Austria; 3 Clinical Institute of Medical and Chemical Laboratory Diagnostics, Medical University of Vienna, Vienna, Austria; Leiden University Medical Center, Netherlands

## Abstract

**Background:**

Mutations in the transcription factor SOX18 are responsible for specific cardiovascular defects in humans and mice. In order to gain insight into the molecular basis of its action, we identified target genes of SOX18 and analyzed one, *MMP7*, in detail.

**Methodology/Principal Findings:**

SOX18 was expressed in HUVEC using a recombinant adenoviral vector and the altered gene expression profile was analyzed using microarrays. Expression of several regulated candidate SOX18 target genes was verified by real-time PCR. Knock-down of SOX18 using RNA interference was then used to confirm the effect of the transcription factor on selected genes that included the guidance molecules ephrin B2 and semaphorin 3G. One gene, *MMP7*, was chosen for further analysis, including detailed promoter studies using reporter gene assays, electrophoretic mobility shift analysis and chromatin-immunoprecipitation, revealing that it responds directly to SOX18. Immunohistochemical analysis demonstrated the co-expression of SOX18 and MMP7 in blood vessels of human skin.

**Conclusions/Significance:**

The identification of *MMP7* as a direct SOX18 target gene as well as other potential candidates including guidance molecules provides a molecular basis for the proposed function of this transcription factor in the regulation of vessel formation.

## Introduction

The SOX family of High Mobility Group (HMG) box transcription factors plays important roles in embryonic development. SOX18, together with SOX7 and SOX17, constitutes the subgroup F within this family. Mutations in SOX18 are the underlying cause of hypotrichosis-lymphedema-telangiectasia (HLT) in humans, a disease that is characterized by sparse hair, bleeding and lymphedema [1]. A similar phenotype (*ragged*) exists in mice and is characterized not only by the name-giving coat defects but also by cardiovascular abnormalities, e.g., edema, cyanosis, dilation, distention and rupture of peripheral blood vessels, including lymphatic defects [2]–[5]. The structure of SOX18 comprises an N-terminal DNA binding domain, a central transactivation domain, and a C-terminal region that contains a recently discovered additional transactivation motif [6]. Several of the cognate mutations in murine Sox18 are located in a short region at the end of the central transactivation and the beginning of the C-terminal part, leading to premature stop codons, and thereby generating dominant-negative (dn) proteins. In contrast to the *ragged* alleles, *Sox18^−/−^* mice show only a mild phenotype [7] which, though it may in part be explained by strain differences, suggests that other transcription factors, possibly members of the Sox subgroup F family, may have redundant function [8]. Indeed, the generation of *Sox18/Sox17* double mutant mice has at least partially supported this view [9], [10].

In the developing mouse embryo, Sox18 is expressed in the allantois and yolk sack blood islands, in the heart, the paired dorsal aortae, in expanding intersomitic and peripheral vessels, in the pancreas, and in nascent vibrissae follicles [5], [11]. In chickens, expression has been described in feather follicles [12], and in humans in fetal brain [13]. In *Xenopus laevis*, sox18 together with sox7 appears to be necessary for cardiogenesis [14]. The combined knock-down of SOX18 and SOX7 in the zebrafish resulted in defective arteriovenous specification [15], [16].

In the adult organism, *SOX18* expression occurs in ventricles and the inter-ventricular septum of the heart [17]. In the vasculature, it is transiently expressed in capillaries within granulation tissue of skin wounds [18]. In atherosclerotic lesions, SOX18 was localized to endothelial cells of the vasa vasorum and intimal neovessels, and also to vascular smooth muscle cells (SMC) in the intima [19]. Expression was also detected in human umbilical vein endothelial cells (HUVEC) and SMC in culture, and found to be necessary for SMC growth in an *in vitro* injury model [19].

Together, the phenotypes observed in human as well as in different experimental model organisms suggest a predominant role of SOX18 in the vasculature, both during development and in the adult. The finding of its expression in a number of tumor cell lines [19], the observation that *ragged* mice show reduced growth of vascularized tumors [20], and the successful inhibition of tumor angiogenesis using cell-permeable dn SOX18 mutants [21] support the view that SOX18 could be a valuable target for interfering with (tumor) angiogenesis. However, despite these important aspects, very little is known about the molecular mechanisms underlying the function(s) of SOX18, i.e. which genes are regulated by the transcription factor. The most prominent one in the context of lymphatic vasculature development is Prox1, however, it requires the venous endothelial-specific nuclear hormone receptor Coup-TFII [2], [22] Two other target genes, *VCAM-1* and the μ-opioid receptor, have been described previously [23], [24], however, these can only partially explain the observed phenotypes. Another one, *Claudin-5*, was reported more recently [25]. Since claudins constitute tight junction strands and maintain cellular barriers, impaired expression could account for the observed edema formation in individuals affected by SOX18 mutations. However, *claudin-5* knock-out mice did not show a corresponding phenotype [26]. Last not least, ROBO4 was found to be transcriptionally regulated by Sox18 in the zebrafish, suggesting a role for Sox18 in vessel guidance [27].

We present here the results of a more global approach aiming at the identification of SOX18 target genes in endothelial cells. Using ectopic SOX18 expression in primary human endothelial cells followed by microarray-based gene expression analysis, we have obtained a comprehensive list of potential target genes. Selected genes were confirmed by real-time PCR and by knock-down experiments. In addition, and to verify the validity of the approach, we have characterized the SOX18-dependent regulation of one of them in more detail, namely matrix metalloproteinase (*MMP7*). The multiple biological effects of this protein during vessel formation may well contribute to the phenotype caused by Sox18 deficiency.

## Materials and Methods

### Plasmids

Human *SOX7*, *SOX17*, and *SOX18* cDNAs were isolated by RT-PCR from HUVEC and cloned into the vector pCMV-myc (Clontech). Promoter fragments for *MMP7* (345 and 196 bps) were isolated by PCR (High Fidelity, Roche), and inserted into the luciferase reporter vector pUBT-Luc [28]. Mutation of the potential SOX18 binding site in the *MMP7* promoter was done using the QuikChange Mutagenesis Kit (Stratagene). The sequence of the primers used for construction is given in Table S1. All constructs were verified by sequencing.

### Cell culture and transfection

HEK293 cells were obtained from ATCC. HUVEC were isolated from human umbilical cords derived from human subjects and propagated as described previously [29]. The use of human umbilical cords for the isolation of HUVEC and the use of skin samples has been approved by the Ethics Commission of the Medical University of Vienna. Written informed consent was obtained from all patients (in the case of umbilical cords, written informed consent was obtained from the parents). Umbilical cords and skin samples were obtained from the Department of Obstetrics and Gynecology, and the Department of Dermatology, respectively, both at the Vienna General Hospital, Medical University of Vienna. HEK293 cells were transfected with the different reporter constructs using the calcium-phosphate method. All transfections were done in triplicates. Luciferase levels were normalized for ß-Gal expression.

### RNA interference

HUVEC were grown in 6-well plates and transfected with siRNAs using polyethylenimine (PEI). The final concentrations in the transfection mix were 250 mM siRNAs and 0.003% PEI. siRNAs directed against either human SOX18, SOX17 or control were purchased from Ambion. Primer sequences are given in Table S1.

### Recombinant adenoviral vectors

*SOX18* was inserted into pIRES-EGFP (Clontech), and then the entire *SOX18*-IRES-EGFP casette was subcloned into the adenoviral transfer vector pACCMVpLpASR+ [30]. The control vector contained only the IRES-EGFP part. Recombinant adenovirus (Adv) was generated by recombination in HEK293 cells as described previously [29]. For transduction, HUVEC were washed twice with serum-free medium and incubated with the adenovirus at a final conc. of 10^8^ pfu/ml for 30 min., followed by replacement with complete medium.

### Microarray analysis

RNA was isolated from non-transduced, Adv-SOX18 and control Adv transduced HUVEC using the RNeasy Mini Kit (Qiagen). Probe labeling and hybridization to HG-U133A 2.0 arrays (Affymetrix) interrogating the expression of 18.400 transcripts, as well as data analysis, was done as described previously [31]. Data conforming to MIAME standards were deposited in the GEO database under the accession nr. GSE8952.

### Real-time PCR

Expression of selected genes was analyzed using a LightCycler with Fast Start SYBR Green I Kit (Roche). Values were normalized for ß2 microglobulin expression. Primers were designed using the program Primer3. Results were confirmed by different sources of RNA. Primer sequences are given in Table S1.

### Electrophoretic mobility shift assay

The Lightshift chemiluminescence EMSA Kit (Pierce) was used with biotinylated oligonucleotides as given in the supplemental materials. Competition experiments were carried out using a 100-fold excess of unlabelled oligonucleotides with either the same, a mutated, or an unrelated sequence. Primer sequences are given in Table S1.

### Chromatin Immunoprecipitation

We used a protocol with two-step cross-linking as decribed [32]. Briefly, 10^7^ cells were pelleted and incubated with 2 mM disuccinylglutarate in PBS for 45 min at room temp, washed with PBS, and crosslinked with 1% paraformaldehyde for 15 min. at RT. The reaction was stopped with 0.125 mM glycine in PBS, cells were washed and suspended in cold lysis buffer containing protease inhibitors (Roche). After 10 min on ice, DNA was sonicated 4× for 25 sec. at 25% power (Branson Sonic Power Company B-12 sonifyer). Debris was removed by centrifugation and the samples stored at −70°C. After pre-clearing with protein A sepharose, 100 µl aliquots were incubated with 2 µg of different anti-SOX18 antibodies (Santa Cruz sc-20100, Thermo Scientific PA1-24474, and Chemicon AB-3207) or rabbit IgG over-night, precipitated with blocked protein A sepharose, and washed with buffer containing increasing salt concentrations. The antibody was eluted from the DNA-protein complex in 1% SDS/0.1 M sodium bicarbonate, the agarose removed by centrifugation, and cross-linking reversed by addition of NaCl to a final conc. of 0.2 M and incubation at 65°C for 4 hours. DNA was purified over Qiagen columns, and analyzed by PCR using the primers given in Table S1. The anti-Sox18 antibody recognized Sox17 10–20 times less efficiently, no detectable cross-reactivity with Sox7 was observed (Figure S2).

### Immunohistochemistry

Serial sections from paraffin-embedded normal human trunk skin were stained with rabbit antibodies against SOX18 (sc-20100; 1∶2500, Santa Cruz), MMP7 (ab4044, Abcam, 1∶500), LYVE-1 (102PA50, Research Diagnostics, Inc., 1∶100), or von Willebrand factor (A0082, Dako, 1∶5000), followed by incubation with biotinylated secondary antibody (BA-1000, anti rabbit IgG, Vector Laboratories, 1∶200). Bound antibodies were visualized by incubation with a streptavidin-HRP conjugate (Dako) and 3-amino-9-ethylcarbazole resulting in a red reaction product. Sections were counterstained with haematoxylin.

## Results

### Identification of SOX18 target genes

Our strategy to identify SOX18 target genes was to ectopically express the transcription factor in primary endothelial cells followed by microarray analysis of the resulting gene repertoire. In order to achieve high transfection efficiency we generated a SOX18 adenoviral vector containing an IRES-EGFP cassette. The control virus contained the IRES-EGFP cassette alone. As judged by EGFP expression, transduction efficiency was close to 100%. As assayed by real-time PCR, expression of Sox18 was approx. 70-fold higher as compared to controls (Figure S1), and did not alter Sox7 and -17 levels. Expression of selected candidate genes was confirmed by real-time PCR. Knock-down of *SOX18* by RNA interference was then used as a complementary approach to verify the dependence of selected genes on the transcription factor.

**Figure 1:**
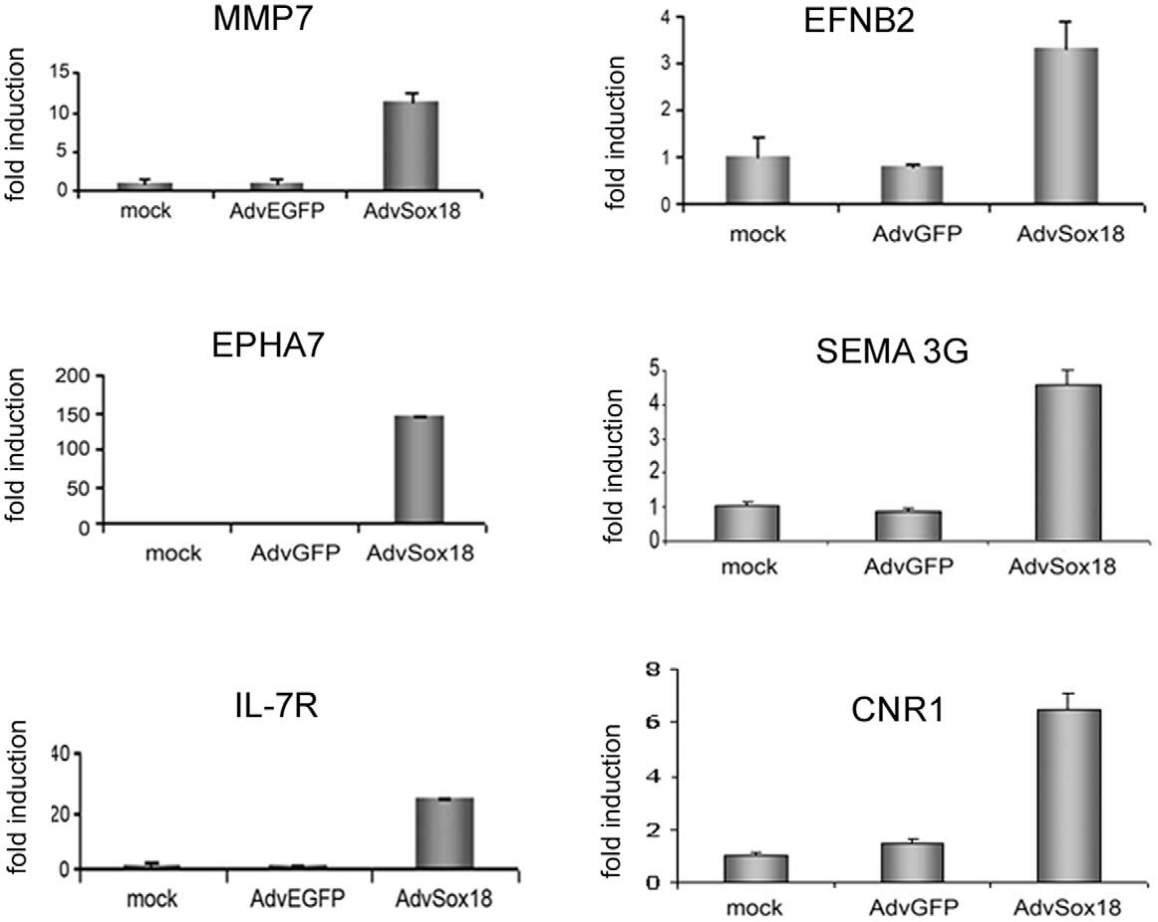
Real-time PCR analysis of SOX18 target genes. RNA was isolated from non-transduced (mock), control virus (AdvEGFP) and SOX18 adenovirus (AdvSOX18) transduced HUVEC and analyzed for the expression of matrix metalloproteinase 7 (*MMP7*), ephrinB2 *(EFNB2), EPHA7*, semaphorin 3G *(SEMA3G)*, interleukin 7 receptor (*IL-7R*), and cannabinoid receptor 1 (*CNR1*). Values were normalized for ß2 microglobulin expression, and are expressed as fold induction compared to control cells.

In order to obtain primary target genes and avoid the isolation of genes that are up-regulated secondary to the induction of others, we harvested the *SOX18* expressing HUVEC at an early time point after transduction. SOX18 protein could be detected as early as 16 hours (not shown). RNA from SOX18-, EGFP-, and non-transduced HUVEC was subjected to microarray analysis. Using a threshold of 3-fold changes, 30 genes were found to be upregulated, and 13 down-regulated (0.23% of the analyzed transcripts, Table 1). Selected genes were further analyzed using real-time PCR, and induction was confirmed for *MMP7*, ephrin-B2 *(EFNB2)*, *EPHA7*, semaphorin 3G (*SEMA3G*), interleukin 7 receptor *(IL-7R)*, cannabinoid receptor 1 *(CNR1)* (Figure 1), and others (Table 1). Whereas the published expression of *VCAM-1* could not be confirmed, both the μ-opioid receptor and claudin-5 were found to be 2-fold increased in the array (not shown). The entire data set has been deposited in the GEO data base (NCBI) under the accession nr. GSE8952.

**Table 1:** Top SOX18 regulated genes.

Accession-nr	Gene symbol	Name	Induction (Sox/EGFP)
Up-regulated			
NM_003925	*MBD4*	methyl-CpG binding domain protein 4	36.90
NM_014421	*DKK2*	dickkopf (*Xenopus laevis*) homolog 2	22.10*
NM_002674	*PMCH*	pro-melanin-concentrating hormone	20.83†
NM_013230	*CD24*	CD24	14.64
NM_004440	*EPHA7*	ephrin receptor A7	11.41*
NM_001321	*CSRP2*	cysteine and glycine-rich protein 2	8.41
NM_002185	*IL7R*	interleukin 7 receptor	5.82*
NM_015148	*PASK*	PAS domain S/T kinase	4.72
NM_012069	*ATP1B4*	ATPase, (Na+)K+ transporting, beta 4	4.65
NM_004392	*DACH*	dachshund (*Drosophila*) homolog	4.63
NM_003467	*CXCR4*	chemokine receptor 4	4.57*
NM_018455	*FLJ13607*	bone marrow protein BM039	4.56
NM_000909	*NPYR*	neuropeptide Y receptor	4.52*
NM_016593	*CYP39A1*	oxysterol 7alpha-hydroxylase	4.40
NM_020469	*ABO*	histo-blood group ABO protein	4.26
NM_001870	*CPA3*	carboxypeptidase A3	4.13
NM_004093	*EFNB2*	ephrinB2	3.81*
NM_015068	*PEG10*	Paternally expressed 10	3.69
NM_016315	*GULP1*	engulfment adaptor PTB domain 1	3.51
NM_004783	*TAO1*	thousand and one amino acid protein kinase	3.39
NM_002423	*MMP7*	matrix metalloproteinase 7	3.38*
NM_006339	*HMG20B*	high-mobility group 20B	3.38
NM_018676	*TMTSP*	thrombospondin module	3.36
NM_000104	*CYP1B1*	cytochrome P450, subfamily I	3.33
NM_006558	*KHDRBS3*	Sam68-like phosphotyrosine protein	3.26
NM_005093	*CBFA2T2*	core-binding factor, alpha subunit 2	3.19
NM_005127	*CLEC2B*	C-type lectin domain family 2B	3.16
NM_016083	*CNR1*	CB1 cannabinoid receptor	3.14*
NM_021624	*HRH4*	histamine H4 receptor	3.14
NM_002245	*KCNK1*	potassium channel, subfamily K, member 1	3.05
NM_020163	*SEMA3G*	Semaphorin3G	2.40*
Down-regulated			
NM_005681	*TAF1A*	TATA box binding protein-associated factor	0.10
NM_017850	*FLJ20508*		0.15
NM_005011	*NRF1*	nuclear respiratory factor 1	0.15
NM_005461	*KRML*	maf-related leucine zipper homolog	0.15
AL049226	*DKFZp564M0916*		0.17
XM_937246	*FLJ13765*	BANP homolog	0.22
NM_018903	*PCDH-alpha12*	VE-cadherin 2 long isoform	0.23‡
NM_001191	*BCL2L1*	Bcl-xS	0.23
NM_000156	*GATM*	guanidinoacetate N-methyltransferase	0.25

*verified by real-time PCR;

† induction by real-time PCR was 1.5-fold;

‡ no change in real-time PCR.

**Figure 2:**
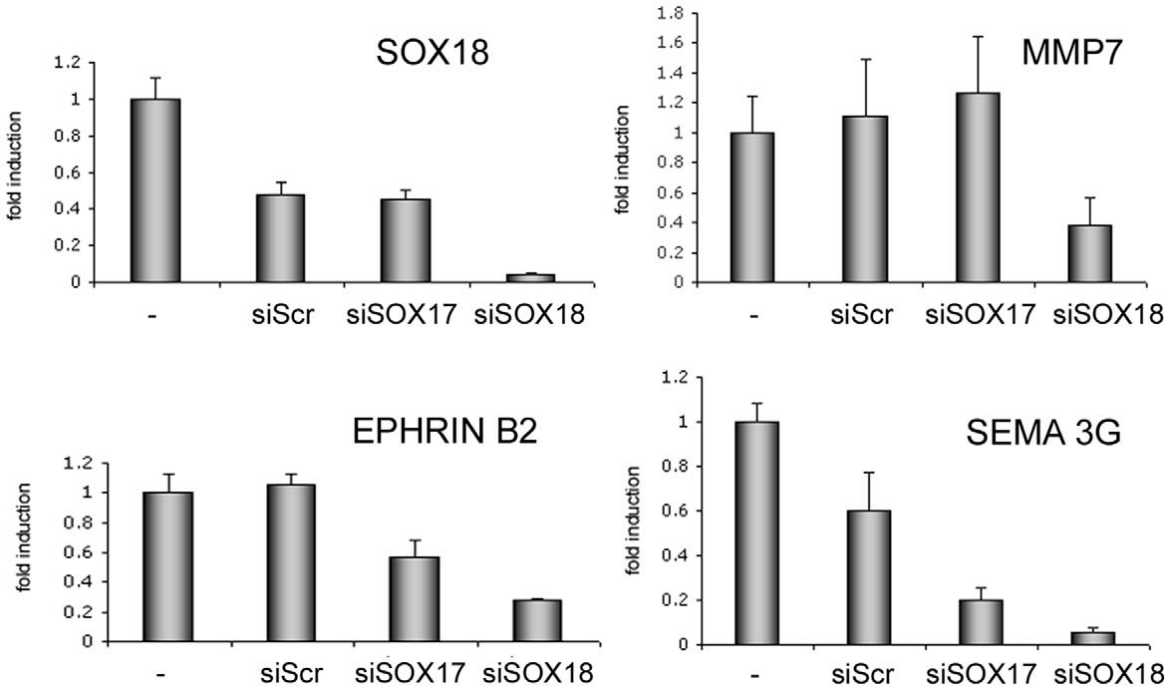
Knock-down of SOX18 diminishes the expression of target genes. HUVEC were transfected with siRNAs directed against *SOX17* or *SOX18*, or with a scrambled control (Scr), and expression of target genes analyzed by real-time PCR. Values were normalized for ß2 microglobulin levels, and are expressed as fold induction compared to control cells.

In a complementary approach we then knocked down SOX18 in HUVEC (and also SOX17 which has been reported to have partially redundant function) using siRNA, and analyzed a subset of genes by real-time PCR. Of these transcripts, *MMP7*, *EFNB2*, and *SEMA3G* were reduced by SOX18 knock-down (Figure 2), the last two also responded to SOX17 knock-down. Two genes (*CNR1* and *EPHA7*) responded weakly, whereas *IL-7R* expression was enhanced (data not shown).

### *MMP7* is a direct target gene of SOX18

Based on its multiple functions in angiogenesis [33]–[37] and clear response to SOX18 in the overexpression and knock-down experiments, we selected *MMP7* for further analysis. A set of experiments was performed to investigate whether it is a primary target or expressed indirectly, i.e., secondary to other regulators that may have been induced by SOX18. A direct target gene is supposed to contain one or more functional SOX18 binding site(s) in the promoter. We therefore analyzed the *MMP7* promoter by reporter gene analysis including mutation of the potential SOX18 binding site, binding of SOX18 *in vitro* using electrophoretic mobility shift assay (EMSA), and *in vivo* using chromatin immunoprecipitation (ChIP).

**Figure 3:**
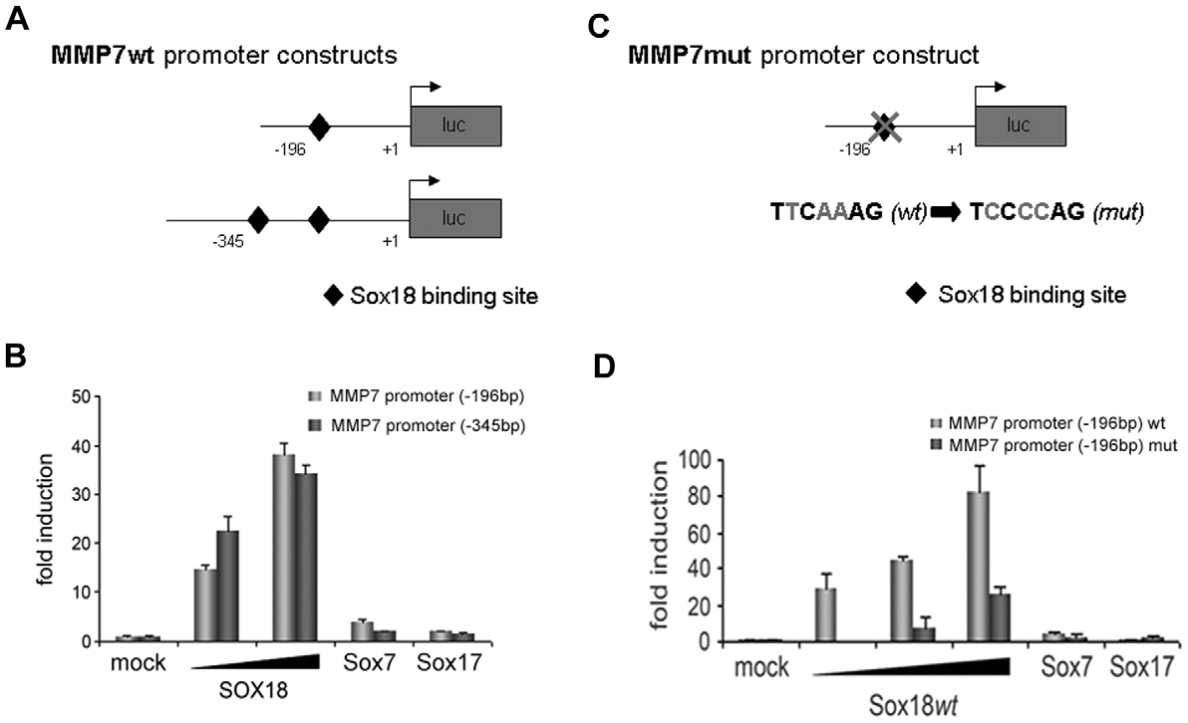
Promoter analysis of *MMP7*. A, Schematic representation of the two *MMP7* promoter-luciferase reporter constructs containing either only the proximal or both potential SOX18 binding sites (black diamonds). B, the wild type (wt) constructs were cotransfected together with increasing amounts of *SOX18, SOX7* or *SOX17* expression vectors into HEK293 cells as indicated. Luciferase values were normalized for ß-Gal expression. C, in the shorter construct, the single potential SOX18 binding site was mutated. D, The short *MMP7* promoter construct containing the mutated SOX18 binding site was analyzed as in (B).

First, bioinformatic analysis using the program TOUCAN [38] revealed the presence of two potential SOX18 binding sites. We isolated two promoter fragments that contained either both or only the proximal site (Figure 3A), and cloned them into the luciferase reporter vector UBT-Luc [28]. Cotransfection of these constructs together with SOX18 resulted in a strong dose-dependent induction of both *MMP7* promoter constructs. In contrast, cotransfected SOX7 or SOX17, the other members of the subgroup F, had only negligible effects (Figure 3B). Since cotransfection experiments showed that the proximal SOX18 binding site is sufficient to induce a strong response only this site was chosen for mutation experiments. Its mutation (Figure 3C) resulted in strongly diminished *MMP7* induction, demonstrating that this site is functional (Figure 3D). Some activity was observed with high amounts of co-transfected Sox18, possibly reflecting non-physiological effects. Furthermore, the short *MMP7* promoter construct did not respond to cotransfected SOX7 or SOX17, the latter being in line with the lack of response to knock-down of SOX17 (Figure 3D).

**Figure 4:**
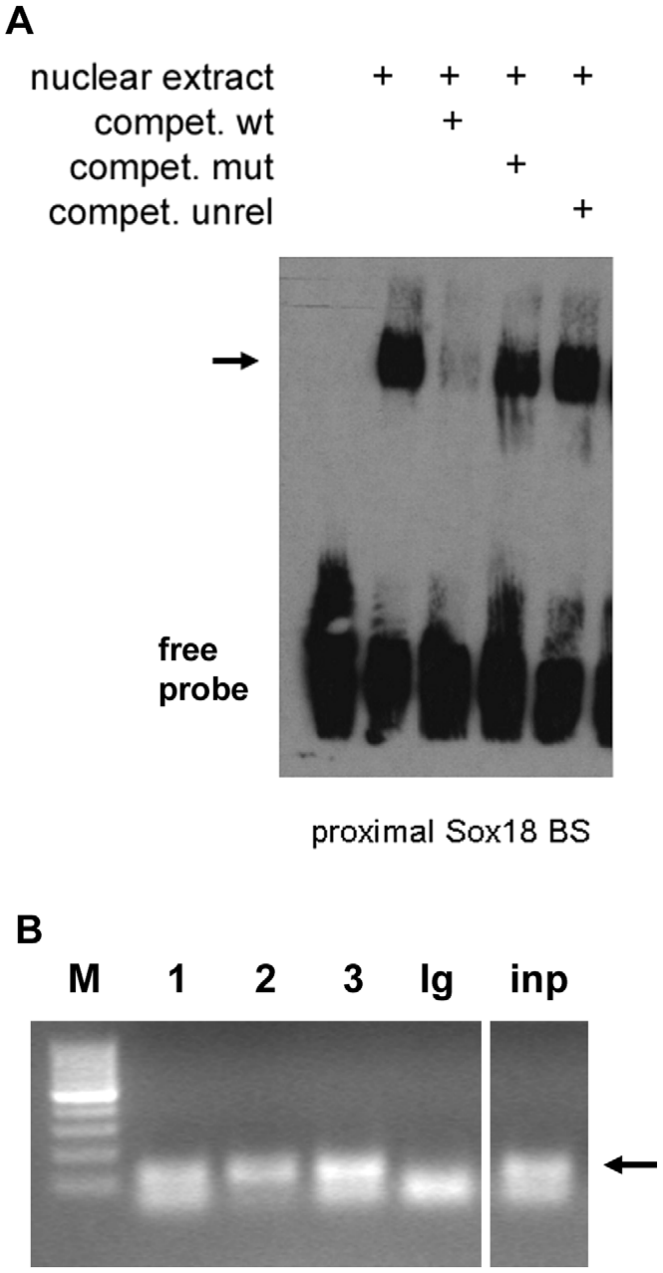
SOX18 binds to the proximal site in the *MMP7* promoter *in vitro* and *in vivo*. A, Electrophoretic mobility shift assay (EMSA). Binding of SOX18 to its proximal site in the *MMP7* promoter was analyzed using nuclear extracts from HUVEC. Specificity of binding was demonstrated by competition experiments using the same (wt), a mutated (mut) or an unrelated (unrel) oligonucleotide as indicated, the specific complex is indicated by an arrow. B, The same site was analyzed by chromatin immunoprecipitation in HUVEC. Lane 1–3: different α-Sox18 antibodies (SantaCruz, Thermo, Chemicon, respectively) as described in Materials and Methods, Ig: IgG control antibody; inp: input, M: 100 bp marker. The 120 bp PCR fragments obtained after amplification ofDNA precipitated by the anti-SOX18 antibodies and input DNA are marked by an arrow.

Next, we determined whether SOX18 binds to the proximal site *in vitro* and *in vivo*. EMSA revealed that nuclear extracts from HUVEC contained a protein binding to the SOX18 binding site, and competition experiments using unlabelled wild type (wt) and mutated oligonucleotides demonstrated the specificity of binding (Figure 4A). However, probably due to technical reasons we were not able to perform a conclusive supershift to directly demonstrate the presence of Sox18 in the complex. HUVEC also express Sox-7 and -17, so in order to assess whether this site was indeed occupied by SOX18 *in vivo*, we performed ChIP. Using primers flanking the proximal SOX18 binding site in the *MMP7* promoter, a specific band could be detected by PCR after precipitation with three different SOX18 antibodies, but not with a control antibody (Figure 4B). Since the Sox18 antibody from SantaCruz recognizes a region with some homology to the other SoxF group members we tested its specificity and found a weak cross-reactivity (1/10–1/20) with Sox17, and none with Sox7 (Figure S2). The Sox18 antibody from Chemicon recognizes an N-terminal 17 aa peptide sequence specific for Sox18. The correct identity of the amplified bands was confirmed by sequencing. Therefore, and in accordance with the reporter and EMSA data, binding of SOX18 to the proximal *MMP7* SOX18 binding site could be demonstrated. We conclude that, using the criteria of *in vitro* and *in vivo* binding and of reporter gene analysis, *MMP7* contains at least one functional SOX18 binding site and represents a direct SOX18 target gene.

### Co-expression of *SOX18* and *MMP7* in skin vessels

**Figure 5:**
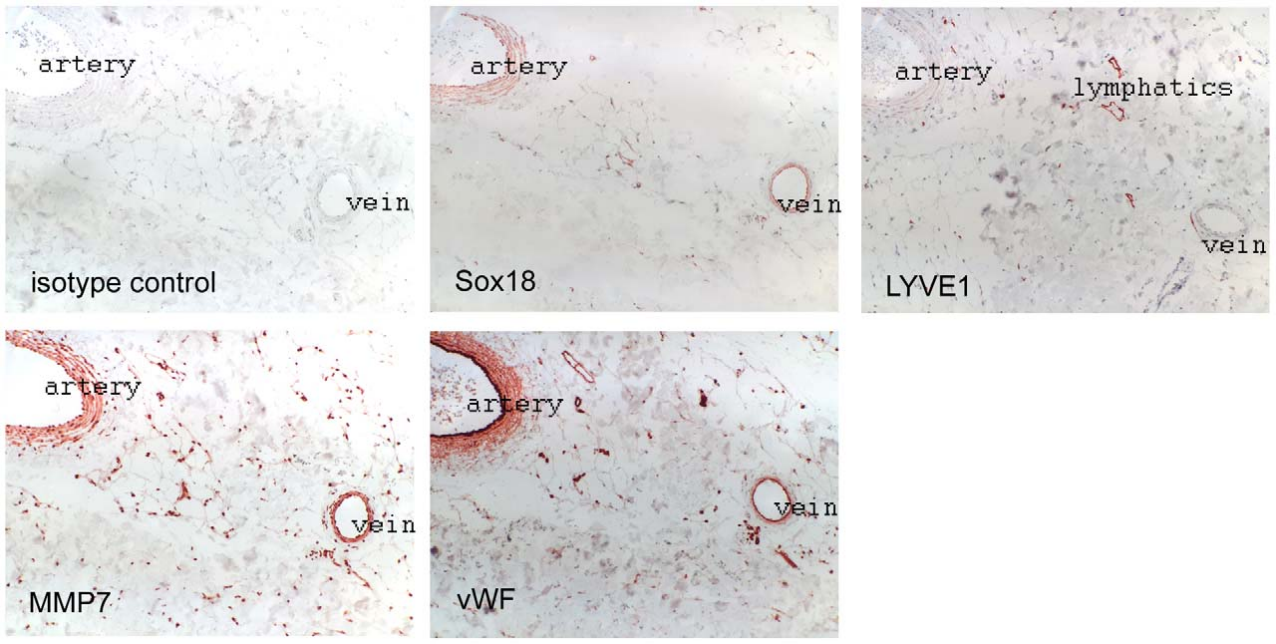
SOX18 and MMP7 are co-expressed in arteries and veins, but not in lymphatic vessels. Serial sections from human skin were analyzed by immunohistochemical staining with the indicated antibodies. Endothelial cells were revealed by von Willebrand factor (vWF) staining, lymphatic vessels by LYVE-1.

*SOX18* has previously been found to be expressed in endothelial and smooth muscle cells [19]. To correlate *SOX18* expression with its target gene we performed immunohistochemical analysis of serial sections of normal adult human skin samples (Figure 5). We found *SOX18* expressed in arteries, veins and some small capillaries, the latter all being LYVE1 negative and thus belonging to the blood vessel tree. *SOX18* positive vessels co-expressed *MMP7*. Moreover, SOX18 stained positive in the muscularis of arteries and veins, and these also expressed *MMP7*. The lack of a complete correlation of SOX18 and MMP7 expression might be due to the fact that MMP7 is also expressed in other cells and in response to stimuli that do not utilize SOX18 (e.g., FGF regulates MMP7 via AP1) [39], or due to diffusion of MMP7 as a secreted protein. Sections were stained with von Willebrand factor to identify all vessels, and LYVE1 for lymphatics. Of note, all lymphatics were negative for SOX18. Furthermore, we have analyzed tissue arrays containing 300 skin samples from basal cell carcinomas of a total of 100 different patients. Similar to the situation in normal skin, all LYVE1 positive lymphatics were negative for SOX18 (data not shown). Although these findings are in contrast with the notion that mutations in *SOX18* affect the lymphatic vasculature [2], it may well be that these alterations occur during development and persist during adulthood without the necessity for continued expression of *SOX18*.

## Discussion

The notion that SOX18 plays an important role in angiogenesis is well supported by the occurrence of mutations in humans and mice that result in vascular (including lymphatic) defects; however, mechanistic insight how this transcription factor exerts its function is still very limited. As a first step to answer this question we have identified SOX18 target genes; one of them, *MMP7*, was analyzed in more detail, and we could show that it represents a direct SOX18 target gene. A recent study described lower levels of Mmp7 and Il-7r in *ragged* mice [40]; while the former is supported by our findings, our data were controversial about the latter: Although expression of *IL-7R* was upregulated upon SOX18 overexpression, it was not reduced, for reasons unknown, upon knock-down in our experimental system.

In vascular cells, multiple functions have been described for MMP7. In general, MMPs degrade a broad variety of proteins, including extracellular matrix proteins. MMP7 substrates include proteoglycans, fibronectin, elastin and casein. SOX18 dependent expression of *MMP7* during angiogenesis could therefore function to promote the sprouting process. However, MMP7 appears to have more functions than just clearing barriers: It has been implicated in ectodomain shedding of several cell surface molecules, including heparin-binding epidermal growth factor precursor, membrane-bound Fas ligand, E-cadherin, and TNFα precursor, all of which are cleaved to release the respective soluble forms [35]. Moreover, VEGF-165, which is bound by connective tissue growth factor, can be released from this inactive complex by MMP7 [34]. Similarly, KC, a member of the CXC family of small chemokines can be liberated from its complex with syndecan-1, thereby generating a KC gradient capable of directing neutrophil migration [33]. These biological activities would suggest a pro-angiogenic function.

However, also anti-angiogenic properties have been attributed to MMP7. First, it can cleave plasminogen and collagen XVIII to generate angiostatin, as well as a 28 kD endostatin-spanning fragment, respectively [41]. Moreover, it cleaves decorin, a proteoglycan that can serve as a reservoir for TGF-ß, a potent anti-angiogenic factor in the extracellular matrix [36]. In a mouse model of corneal wound healing, MMP7 served as an inhibitor of corneal avascularity [37]. Therefore, the net outcome of SOX18 dependent *MMP7* expression cannot be predicted, and may depend on the specific site and context of the angiogenic process.

Beside *MMP7*, we have identified several other potential target genes of SOX18. Combining SOX18 overexpression with the more stringent criterion of SOX18 knock-down, *EFNB2* and *SEMA3G* turned out to be reliable candidates. It should be noted that *MMP7* responded only to SOX18 knock-down, whereas the other two were dependent (however, to a lesser degree) on SOX17 levels. Both EFNB2 and SEMA3G serve as guidance molecules in endothelial cells. EFNB2 and its counter-receptor EPHB4 are expressed on developing arteries and veins respectively, and repulsive signaling between the two molecules has been suggested to maintain the boundaries between the two types of vessels during network formation [42]. The identification of *EFNB2* as a SOX18 target fits well to the recent observations that SOX18 knock-down in the zebrafish results in impaired arteriovenous specification [15], [16]. Less information exists for SEMA3G, a member of larger family of phylogenetically conserved secreted and membrane-bound proteins. SEMA3G is a secreted molecule that binds to Neuropilin-2, but not Neuropilin-1, receptors that are also bound by certain VEGF forms. In neuronal development, SEMA3G induced the repulsion of sympathetic, but not dorsal root ganglion axons, indicating that it can selectivity repel specific types of axons [43]. Semaphorin class 3 family members have been suggested as modulators of angiogenesis [44]. Together, and also considering the additional upregulation of *EPHA7* (which, however, did not clearly respond to SOX18 knock-down), suggests that one function of SOX18 may be the control of directed vessel formation. For reasons unknown, we did not identify VCAM-1, a published Sox18 target gene; one explanation could be that our experimental setup of Sox18 overexpression as a primary screen would allow solely the identification of genes for which Sox18 is alone sufficient, but not those where it is necessary (in combination with other factors). We also did not find Prox1 in HUVEC, however Sox18 could induce its expression in endothelial progenitor cells (not shown). This is consistent with the finding that Coup-TFII has been identified as a cofactor for Prox1 initial expression and maintainance [22].

Since a single transcription factor usually participates in the regulation of several genes, mutations that impair its function can be expected to affect a broad spectrum of genes and result in a complex phenotype. Diminished *MMP7* expression as a result of a mutated SOX18 may already affect several aspects of angiogenesis, both in the developing as well as the adult organism. In addition, the identification of EFNB2 and SEMA3G suggests a role for SOX18 in vessel guidance.

Taken together, the present work represents a functional way to identify potential SOX18 target genes. With the identification of *MMP7* and performed functional analysis, the direct regulatory relationship between SOX18 and *MMP7* was demonstrated. Future studies will focus on other potential target genes identified by this approach, to further investigate the role of SOX18 and its target genes in vessel formation. With the more detailed characterization of additional direct Sox18 target genes a better understanding of the molecular mechanisms of SOX18 in the *ragged* phenotype in mice and the HLT syndrome in humans will be gained.

## Supporting Information

Figure S1 **Overexpression of Sox18 by a recombinant adenovirus.** HUVEC were either mock transfected or transfected with a recombinant adenovirus for expression of GFP or of Sox18 as indicated, and mRNA isolated 16 hours later and analyzed by real-time PCR for expression of Sox7, -17 and -18. Values were normalized to ß2-microglobulin.
(TIF)

Figure S2 **Crossreactivity of Sox18 antibody.** A, HEK293 cells were transfected with myc-tagged expression vectors for Sox7, -17, and -18, or empty vector (EV), and protein extracts analyzed by Western blotting using anti-myc and anti-Sox18 (SantaCruz). B, repetition of the experiment using Sox17 and -18 and different times of exposure.
(TIF)

Table S1 Primers used for constructs, real-time PCR, EMSA, ChIP, and knock-down experiments.
(DOC)
