# Supplementary figures and images for: The Transcription Factor SOX18 Regulates the Expression of Matrix Metalloproteinase 7 and Guidance Molecules in Human Endothelial Cells
Source: PLoS One. 2012 Jan 23;7(1):e30982. doi: 10.1371/journal.pone.0030982 (PMC3264645; doi:10.1371/journal.pone.0030982)

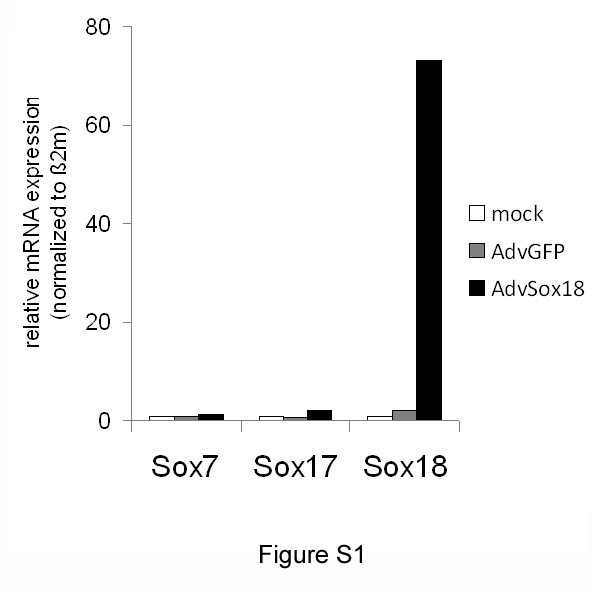

Supplement: Figure S1 — Overexpression of Sox18 by a recombinant adenovirus. HUVEC were either mock transfected or transfected with a recombinant adenovirus for expression of GFP or of Sox18 as indicated, and mRNA isolated 16 hours later and analyzed by real-time PCR for expression of Sox7, -17 and -18. Values were normalized to ß2-microglobulin. (TIF) [file pone.0030982.s001.tif]

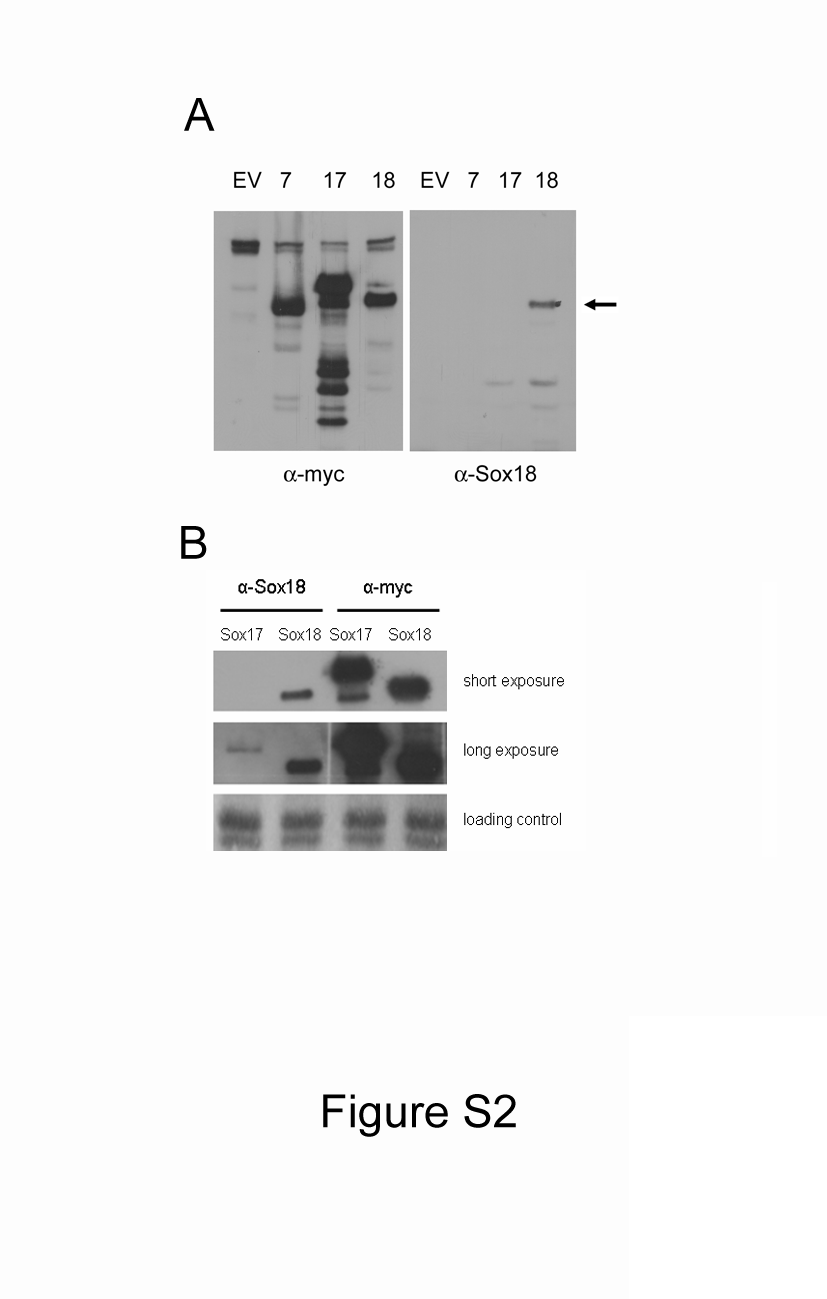

Supplement: Figure S2 — Crossreactivity of Sox18 antibody. A, HEK293 cells were transfected with myc-tagged expression vectors for Sox7, -17, and -18, or empty vector (EV), and protein extracts analyzed by Western blotting using anti-myc and anti-Sox18 (SantaCruz). B, repetition of the experiment using Sox17 and -18 and different times of exposure. (TIF) [file pone.0030982.s002.tif]
