# Supplementary material for: The Transcription Factor SOX18 Regulates the Expression of Matrix Metalloproteinase 7 and Guidance Molecules in Human Endothelial Cells
Source: PLoS One. 2012 Jan 23;7(1):e30982. doi: 10.1371/journal.pone.0030982 (PMC3264645; doi:10.1371/journal.pone.0030982)
Supplement: Table S1 — Primers used for constructs, real-time PCR, EMSA, ChIP, and knock-down experiments. (DOC) [file pone.0030982.s003.doc]

Supplemental Material

Primers for Sox expression constructs

| SOX18 for | ACGCGTCGACCATGCAGAGATCGCCG |
| --- | --- |
| SOX18 rev | GAAGATCTCTAGCCGGAGATGCA |
| SOX17 for | ACGCGTCGACCATGAGCAGCCCGGAT |
| SOX17rev | GAAGATCTTCACACGTCAGGATAGTT |
| SOX7 for2 | ACGCGTCGACCATGGCTTCGCTGCTG |
| SOX7rev | CTATGACACACTGTAGCTG |
| SOX18dn rev | CGGGATCCGGCCTGCTTCTTCCTG |

Primers for promoter constructs

| MMP7for1long | AAAAGCGGCCGCTGGTACCATAATGTCCTG |
| --- | --- |
| MMP7for2short | AAAAGCGGCCGCCGATGTAATACTTCCTCG |
| MMP7rev | GGACTAGTCTCGAATGTGGAAATAGG |
| MMP7s mut1for | GAAAAAATAACACATACTTCCCCAGTTCTGTAGACTC |
| MMP7s mut1rev | GAGTCTACAGAACTGGGGAAGTATGTGTTATTTTTC |

Primers for real-time PCR

| IL7Rfor | TCGATCCATCCCTGATCACT |
| --- | --- |
| IL7Rrev | GGAGACTGGGCCATACGATA |
| ephrinB2for | CTGCTGGATCAACCAGGAAT |
| ephrinB2rev | GATGTTGTTCCCCGAATGTC |
| EphA7for | TGCAGTGCAGAAGGAGAATG |
| EphA7rev | GGTCAGATGGAGCCCTGTAA |
| dickkopf for | TGATGGTGGAGAGCTCACAG |
| dickkopf rev | TGGCAATACCTCCCAACTTC |
| MMP7 for | TGTATGGGGAACTGCTGACA |
| MMP7 rev | GCGTTCATCCTCATCGAAGT |
| CXCR4 for | CCAGTTTCAGCACATCATGG |
| CXCR4 rev | GAGTCGATGCTGATCCCAAT |
| VEcadherin for | CGTTACTCTGCCTCCCTGAG |
| VEcadherin rev | AAGGTCCAGCTGTTGCTGTT |
| sema3G for3 | TCATTTTCCTGGGGACTGAC |
| sema3G rev3 | TGCCGTAAGTCTCACATTGG |
| NPYR fo | TTGGTGCTGCAGTATTTTGG |
| NPYR rev | GTGGTTGCAGGTAGCAATGA |
| PMCH fo | GCTCCTTCCCTGGAACAATA |
| PMCH rev | AGCCAGATTCAGTGGCAGAC |
| CNR1 fo | TCCTAGATGGCCTTGCAGAT |
| CNR1 rev | CTCCCCACACTGGATGTTCT |

Primers for EMSA

| MMP7 EMSA + | CACATACTTTCAAAGTTCTGTAGAC |
| --- | --- |
| MMP7 EMSA - | GTCTACAGAACTTTGAAAGTATGTG |
| MMP7 EMSAmut + | CACATACTTCCCCAGTTCTGTAGAC |
| MMP7 EMSAmut - | GTCTACAGAACTGGGGAAGTATGTG |

Primers for ChIP

| MMP7 chip short for | CAATAACGATGTAATACTTCCTCG |
| --- | --- |
| MMP7 chip short rev | GCCTCGAATGTGGAAATAGG |

siRNAs

|  | sense | antisense |
| --- | --- | --- |
| Sox18 | GGGUUACAUUUUUGAAGCATT | UGCUUCAAAAAUGUAACCCTG |
| Sox17 | GGCCAGAAGCAGUGUUACATT | UGUAACACUGCUUCUGGCCTG |
| control | Ambion Silencer® Negative control #1 (AM4611) |  |
